# Community-acquired *Escherichia coli* meningitis with ventriculitis in an adult—a rare case report

**DOI:** 10.1186/s40560-018-0332-6

**Published:** 2018-09-24

**Authors:** Rajesh Kasimahanti, Sai Kandraju Satish, Mridu Anand

**Affiliations:** 1 Department of Critical Care Medicine, Yashoda Hospitals, Alexander Road, PIN: 500003, Secunderabad, Telangana India; 2 Department of Neurology, Yashoda Hospitals, Secunderabad, India; 3 Department of Microbiology, Yashoda Hospitals, Secunderabad, India

**Keywords:** Community-acquired meningitis, Ventriculitis, *E. coli*, CSF leak

## Abstract

**Background:**

Community-acquired gram-negative bacillary meningitis is rare to occur without preexisting conditions like trauma, organ dysfunction, and immunocompromised state, and very few case reports with *Escherichia coli* have been described in literature till now. Presence of ventriculitis along with meningitis makes the incidence further sparse.

**Case presentation:**

A review of literature identified a total of only 45 community-acquired *E. coli* meningitis from 1945 till to date. Here, we have described a case of community-acquired *E. coli* meningitis with ventriculitis in an adult with past history of completely repaired CSF leak secondary to trauma nearly 23 years ago, without current radiological evidence of persistent CSF leak and therefore described as spontaneously acquired. Post-contrast T1 images of MRI were suggestive of subtle ependymal enhancement of ventricles, and patient was treated in lines of ventriculitis. Initial CSF was suggestive of acute pyogenic meningitis, and the organism grown was pan-sensitive *E. coli*. Patient was treated with antibiotics according to the culture sensitivity pattern and was given a prolonged course of 6 weeks of antibiotic therapy in view of ventriculitis.

**Conclusion:**

Community-acquired *E. coli* meningitis with possible ventriculitis in adults is described as a rare entity and is likely to be underrated and under-recognized.

## Background

Gram-negative bacilli (GNB) are a relatively uncommon cause of community-acquired meningitis in adults ranging from 0.7 to 7% across the world [1]. Most of the GNB meningitis occurs after a neurosurgical procedure, within a month of head trauma, in the presence of a neurosurgical device or any cerebrospinal fluid (CSF) leak syndrome. They represent 75% of the cases of nosocomial gram-negative bacillary meningitis [2]. The reported annual incidence of spontaneous GNB meningitis in adults is around 2 cases per 100,000 adults. The highest reported incidence is around 8.7%, of which, *Escherichia coli* (*E. coli*) represents around 41.9% [3]. Post-traumatic CSF leak predisposing GNB meningitis usually presents within a month of trauma, and the risk reduces after the repair of leak without any residual defect. At the same time, presence of ventriculitis along with community-acquired meningitis is much rare. To the best of our knowledge, there were no case reports hitherto of *E. coli* meningitis with ventriculitis. Here, we are describing a case of community-acquired spontaneous *E. coli* meningitis with ventriculitis which presented 23 years after complete repair of CSF leak for head trauma.

## Case presentation

A 56-year-old male patient with no known comorbidities presented to the emergency department with history of fever and headache for past 2 days with sporadic episodes of projectile vomiting, irrelevant talk, and poor sensorium for a day. On admission, the patient manifested fever (39 °C), a heart rate of 104 beats per minute, blood pressure of 130/60 mmHg, respiratory rate of 30/min, and capillary blood glucose of 140 mg/dl. His Glasgow Coma Scale (GCS) score was 9, with respective scores of 5, 2, and 2 for motor, eye, and verbal responses. There were no signs of meningeal irritation or focal neurological deficit. Clinical examination was otherwise normal except for a scar over the anterior bi-frontal region which was a surgical incision. On further inquiry, he had an accidental fall from a two-story building 23 years ago, causing a persistent CSF leak from the nose (rhinorrhea). He underwent complete repair for the CSF leak without any residual defect. There were no further hospitalizations for the past 23 years with symptoms suggestive of meningitis.

**Fig. 1:**
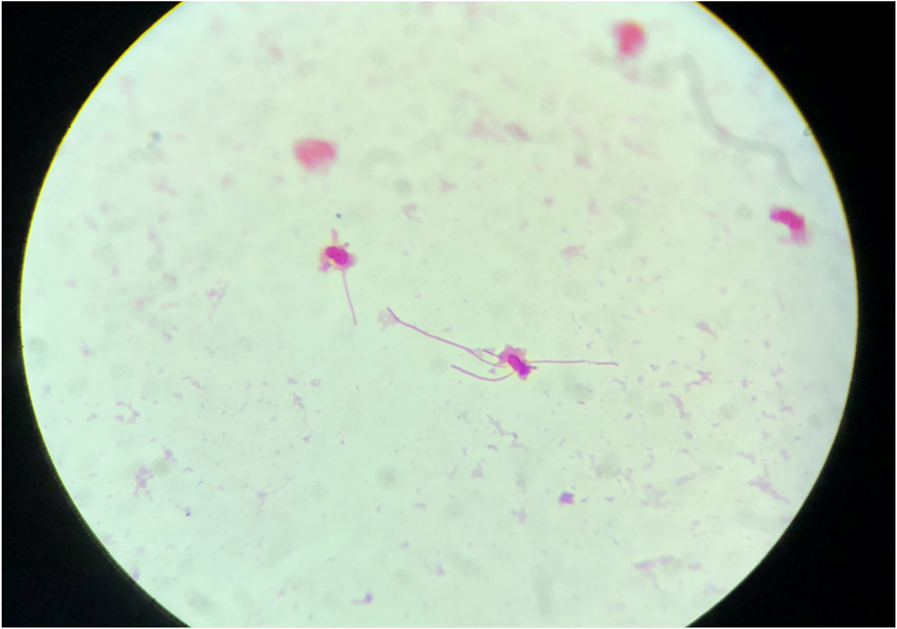
Gram-negative filamentous rods

**Fig. 2:**
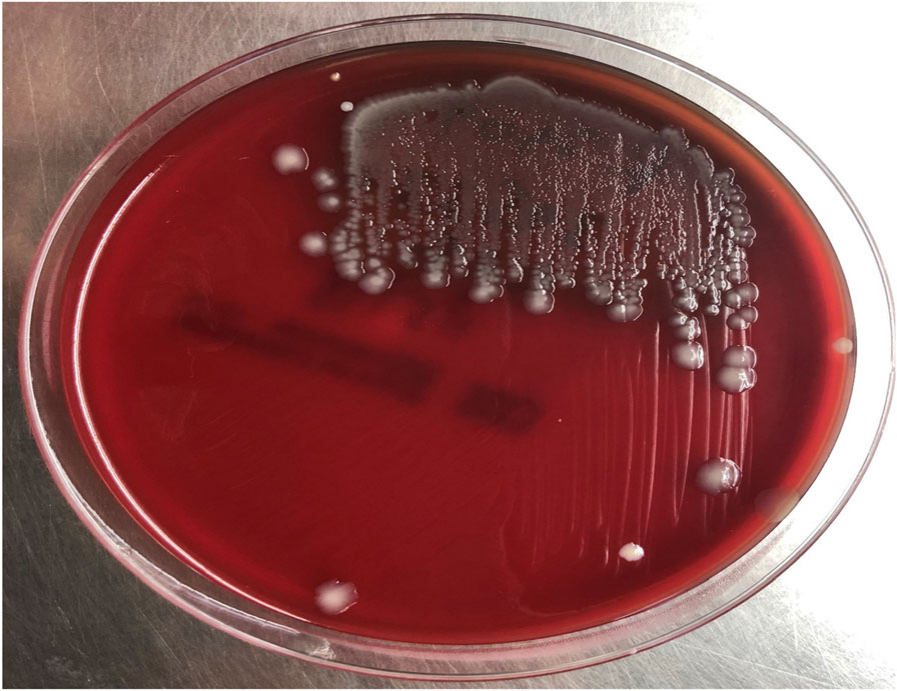
Non-hemolytic colonies on blood agar

**Fig. 3:**
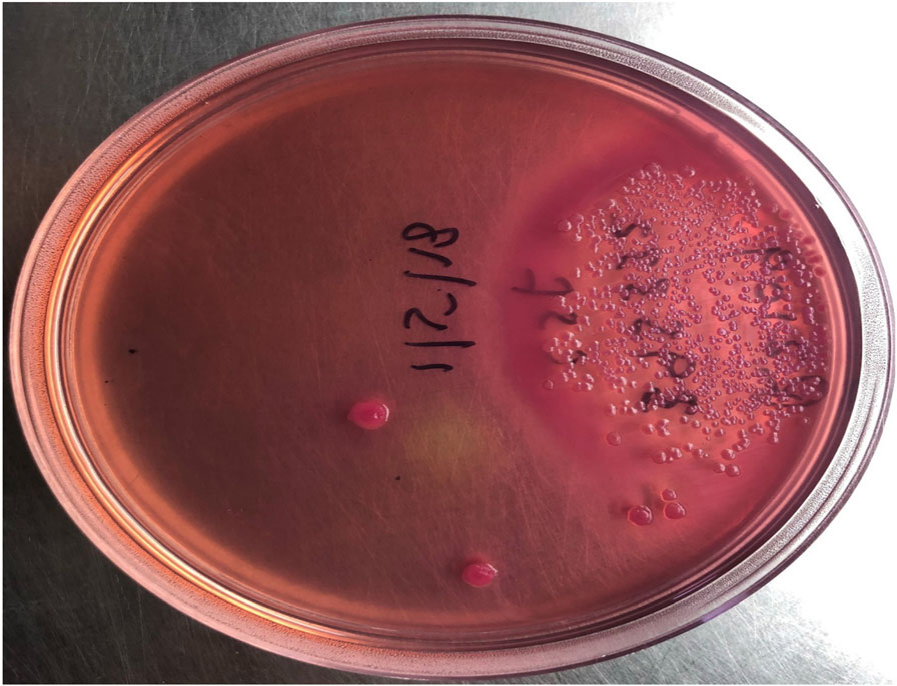
Lactose-fermenting colonies on MacConkey agar

His computerized tomography (CT) scan showed cystic gliosis changes in the left frontal lobe which was communicating with the frontal horn of ipsilateral lateral ventricle. CSF analysis of the patient revealed hypoglycorrachia (< 20 mg/dl with corresponding blood sugar of 140 mg/dl), elevated protein > 300 mg/dl, and neutrophil-predominant pleocytosis (total cells 360/mm^3^ with 96% polymorphs) suggestive of acute pyogenic meningitis. He was started empirically on ceftriaxone 100 mg/kg/day in two divided doses, amoxycillin 100 mg/kg/day in four divided doses, and acyclovir 45 mg/kg/day in three divided doses. Initial peripheral blood WBC count was 14,760 with 85% neutrophils and serum lactate levels of 5.35 mmol/l. His renal and liver function tests were normal. Antibiotics (ceftriaxone, amoxycillin) were continued, and acyclovir was stopped as there were filamentous gram-negative rods in gram staining of the CSF, which was a rare morphology to be identified (Fig. 1). Further, the sample was plated onto blood agar, MacConkey agar, and chocolate agar and incubated aerobically at 37 °C. Non-hemolytic colonies were formed on blood agar (Fig. 2) which were lactose-fermenting colonies on MacConkey agar (Fig. 3). The organism was identified to be *Escherichia coli* by Vitek 2 Compact (Biomerieux), and spectrum of antibiotic sensitivity was described in Table 1.

**Table 1:** Spectrum of Antimicrobial sensitivity

Organism: *Escherichia coli*		
Antibiotic	MIC	Sensitivity
Ampicillin	> 32	R
Cefuroxime	= 4	S
Ceftriaxone	< 1	S
Cefipime	< 1	S
Meropenem	< 0.25	S

### Further course

**Fig. 4:**
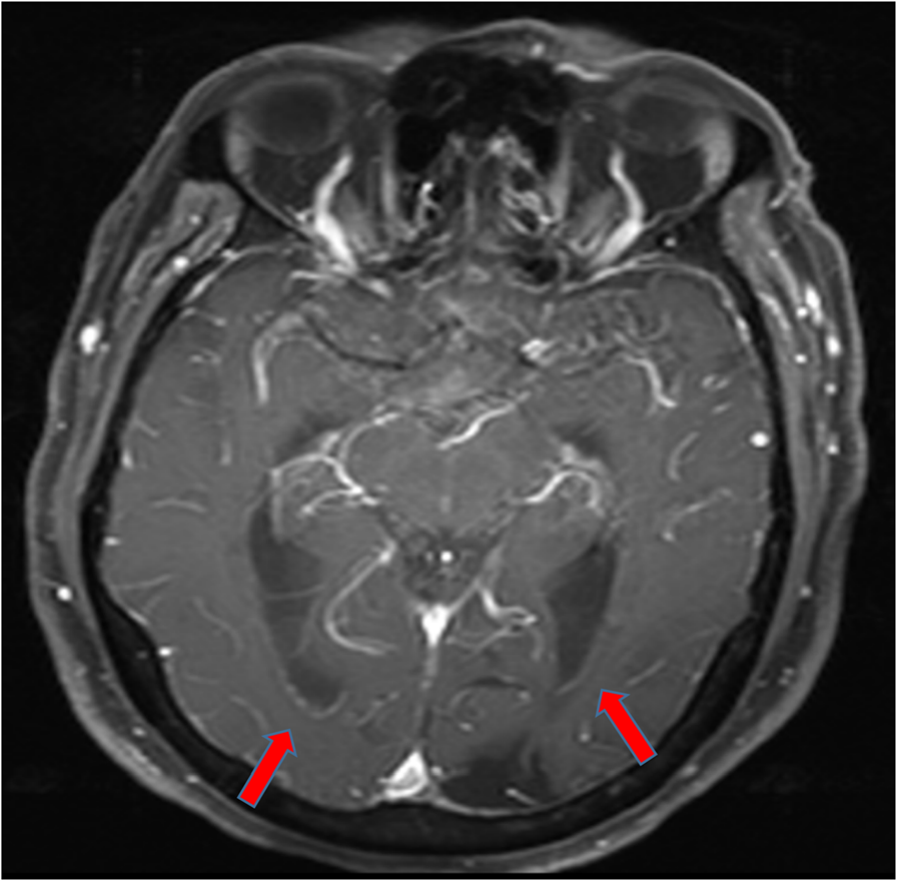
Post-gadolinium T1 image showing subtle ventricular ependymal enhancement (arrow) in occipital horns

**Fig. 5:**
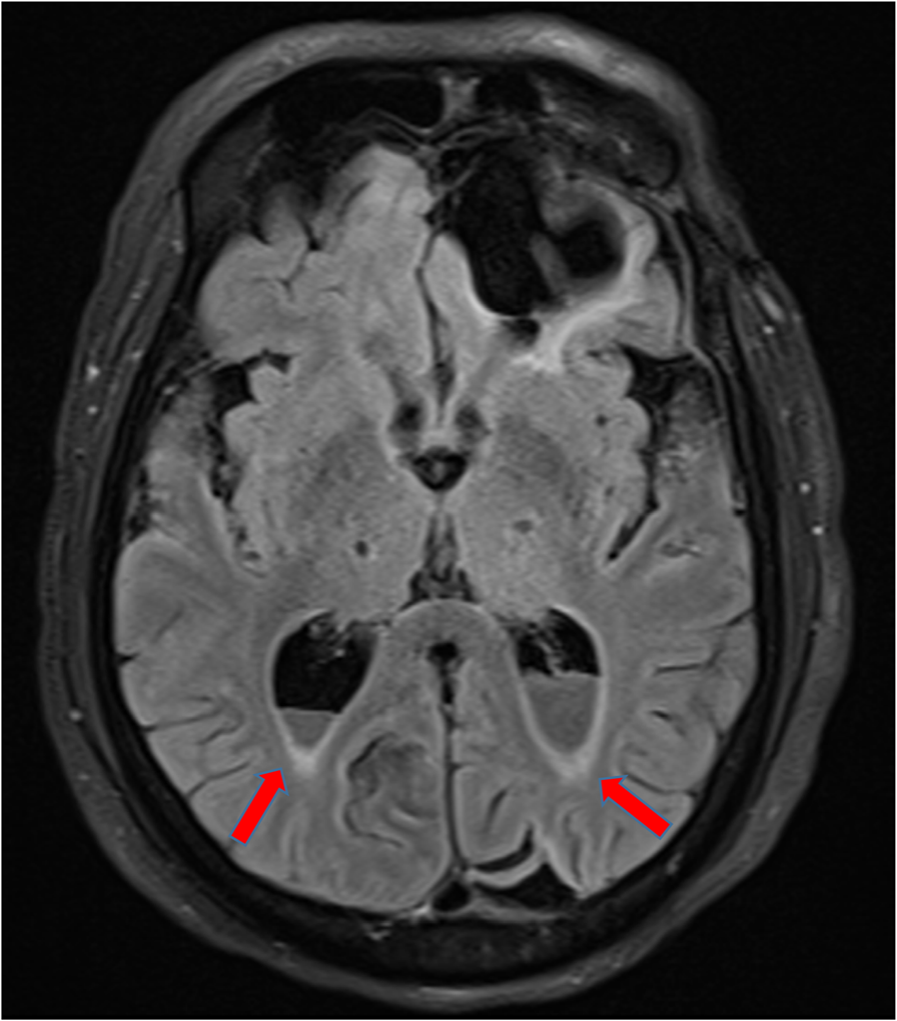
Post-gadolinium T2 FLAIR showing subtle ventricular ependymal enhancement and exudates (arrow) in occipital horns

**Fig. 6:**
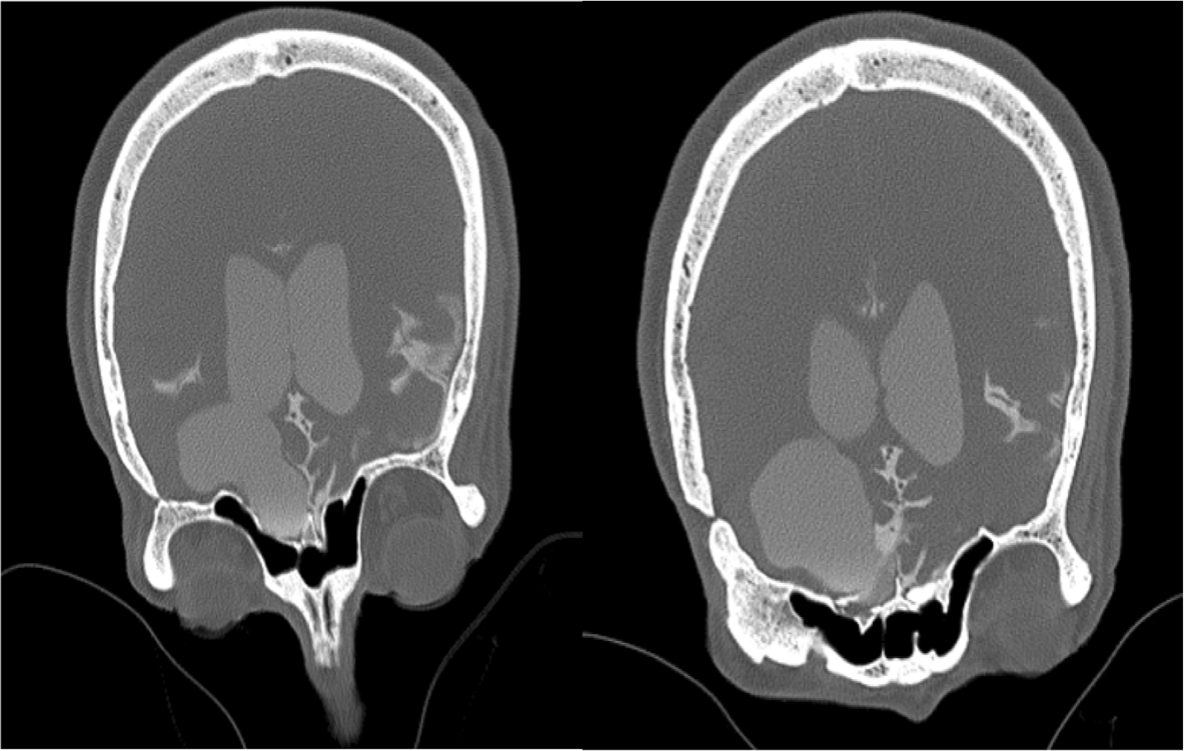
CT cisternography showing no contrast leak into paranasal sinuses

In accordance with the culture and sensitivity pattern, ceftriaxone was continued and amoxycillin was stopped. He was afebrile for the next 48 h, and his sensorium improved. Further work up was done to identify the source of seeding of *E. coli* to the meninges. Urine culture and blood cultures that were sent prior to initiation of antibiotics did not show any bacterial growth, and stool microscopy was negative for ova and cysts. CT scan of the abdomen revealed a non-obstructive renal calculus of 6 mm × 6mm at interpole of the left kidney without any signs of pyelonephritis. After the initial improvement, patient again had a drop in sensorium without any localizing signs. Magnetic resonance imaging (MRI) of the brain with contrast study was done to rule out secondary complications associated with meningitis like vascular infarcts or hydrocephalus. There was post-contrast enhancement of leptomeninges and subtle enhancement of ventricular wall and posterior fossa cisternal spaces in post-contrast T1 sequence, and the same findings along with exudates in the ventricles in T2 fluid attenuated inversion recovery (FLAIR) images, which were suggestive of ventriculitis (Figs. 4 and 5). CT cisternogram was performed to check for residual CSF leak at the past surgical site, in which there was an opacified density in the left frontal region representing a porencephalic cyst communicating with the left frontal horn. There was no CSF leak into the paranasal sinuses or nasal cavity (Fig. 6).

In the CSF analysis repeated on day 7 of antibiotic therapy, there was improvement in cell count (total cell count of 26 with 100% of lymphocytes) and cultures were sterile. He was discharged from the hospital on day 10 and advised to continue intravenous antibiotics for 6 weeks in view of ventriculitis. On further follow-up, the patient has improved, with no neurological deficit and antibiotics have been stopped at the end of the sixth week.

## Discussion and conclusions

Community-acquired spontaneously occurring *E. coli* meningitis in adults is rare, and the recent report by Bichon et al. showed a total of 45 cases of the same from 1945 to 2017 with an average report of one case per year across the world [1]. Various risk factors have been identified for the same. Among them, alcoholism with cirrhosis, uncontrolled diabetes, disseminated strongyloidiasis, HIV, and chronic organ dysfunction are the most common [1]. Previously reported cases identified the primary focus of *E. coli* from the urinary tract or GI tract, and a rare case report has identified the source as a retropharyngeal abscess [4]. In our case, the patient underwent a neurosurgical procedure for CSF leak two decades ago. He neither had symptoms of a leak nor an evidence of CSF leak could be demonstrated during this admission. Hence, this meningitis cannot be labeled as postsurgical meningitis. Presence of subtle features of ventriculitis on imaging makes it further uncommon, especially when it is community-acquired. Preantibiotic era has shown presence of ventriculitis at the end of the first week in pathological examinations, but the current incidence is very low [5] and the literature was silent especially on community-acquired *E. coli* meningitis. At the same time, we could not find the primary source for *E. coli* in our case. This could be explained by the prior oral antibiotics taken at the time of onset of fever. Presence of filamentous gram-negative rods in microscopy also strengthens the prior antibiotic exposure [6]. We tried to look into other rare causes like strongyloidiasis and chronic organ insufficiency which were absent in our patient. In this patient, possible source of *E. coli* could be from the urinary tract as there was a renal calculus. However, urine culture was negative. Ruling out all possible sources of *E. coli*, the infection might be community-acquired in our case.

Most commonly, wild type of *E. coli* causes meningitis up to 20% of cases [1]. Emergence of extended spectrum betalactamase (ESBL) production at community level in *E. coli* is a concern. Bichon et al. reported that up to 7% of community-acquired infections with *E. coli* show ESBL production and 9% of *E. coli* show broad spectrum betalactamase production with resistance to penicillin group of antibiotics [1]. In our case report, it is a broad spectrum betalactamase producer with sensitivity to third-generation cephalosporins. While there are precise guidelines for the management of ventricular-catheter-related infections [5], we found neither recommendations nor expert advice regarding the optimal regimen or duration of the management of the treatment of primary bacterial ventriculitis. A 6- to 12-week duration of treatment, similar to recommendation for brain abscesses [7], was deemed essential in ventriculitis. However, given the severity of disease, a longer duration of antibiotics has to be given if the tolerance is acceptable.

Mortality with community-acquired *E. coli* meningitis is significant, and it ranges from 50 to 90%, reaching to 85% in disseminated Strongyloidiasis and up to 100% in case of cirrhosis. Presence of multi-organ dysfunction worsens the chances of recovery. But in the recent review, Bichon et al. identified mortality as low as 47% with possible chances of recovery.

In conclusion, community-acquired *E. coli* meningitis with ventriculitis in adults is described as a rare entity and is likely to be underrated and under-recognized. Newer risk factors identified, like urinary tract infection, digestive tract disorders, disseminated Strongyloidiasis, have to be verified in all proven cases. Emergence of ESBL production in wild type of *E. coli* is a concern with over the counter availability of antibiotics. Finally, the prognosis of meningitis with ventriculitis is determined by the diagnosis, timeliness, and adequate duration of antibiotic therapy.

## Abbreviations

CSF: Cerebrospinal fluid
CT: Computerized tomography
E. coli: *Escherichia coli*
ESBL: Extended spectrum betalactamase
FLAIR: Fluid-attenuated inversion recovery
GCS: Glasgow coma scale
GNB: Gram-negative bacilli
MRI: Magnetic resonance imaging
WBC: White blood cells
